# Combinatorial Virtual Screening Revealed a Novel Scaffold for TNKS Inhibition to Combat Colorectal Cancer

**DOI:** 10.3390/biomedicines10010143

**Published:** 2022-01-10

**Authors:** Chun-Chun Chang, Sheng-Feng Pan, Min-Huang Wu, Chun-Tse Cheng, Yan-Rui Su, Shinn-Jong Jiang, Hao-Jen Hsu

**Affiliations:** 1 Department of Laboratory Medicine, Hualien Tzu Chi Hospital, Buddhist Tzu Chi Medical Foundation, Hualien 97004, Taiwan; 101353110@gms.tcu.edu.tw; 2 Department of Laboratory Medicine and Biotechnology, College of Medicine, Tzu Chi University, Hualien 97004, Taiwan; 107312149@gms.tcu.dedu.tw (M.-H.W.); 107312156@gms.tcu.dedu.tw (Y.-R.S.); 3 Department of Biochemistry, School of Medicine, Tzu Chi University, Hualien 97004, Taiwan; 107330102@gms.tcu.edu.tw; 4 Department of Life Sciences, College of Medicine, Tzu Chi University, Hualien 97004, Taiwan; 107711124@gms.tcu.edu.tw

**Keywords:** TNKS inhibitor, virtual screening, pharmacophore, Wnt signaling pathway, docking, β-catenin

## Abstract

The abnormal Wnt signaling pathway leads to a high expression of β-catenin, which causes several types of cancer, particularly colorectal cancer (CRC). The inhibition of tankyrase (TNKS) activity can reduce cancer cell growth, invasion, and resistance to treatment by blocking the Wnt signaling pathway. A pharmacophore search and pharmacophore docking were performed to identify potential TNKS inhibitors in the training databases. The weighted MM/PBSA binding free energy of the docking model was calculated to rank the databases. The reranked results indicated that 26.98% of TNKS inhibitors that were present in the top 5% of compounds in the database and near an ideal value ranked 28.57%. The National Cancer Institute database was selected for formal virtual screening, and 11 potential TNKS inhibitors were identified. An enzyme-based experiment was performed to demonstrate that of the 11 potential TNKS inhibitors, NSC295092 and NSC319963 had the most potential. Finally, Wnt pathway analysis was performed through a cell-based assay, which indicated that NSC319963 is the most likely TNKS inhibitor (pIC_50_ = 5.59). The antiproliferation assay demonstrated that NSC319963 can decrease colorectal cancer cell growth; therefore, the proposed method successfully identified a novel TNKS inhibitor that can alleviate CRC.

## 1. Introduction

Colorectal cancer (CRC) is the second deadliest cancer in the world and 1.93 million new cases were diagnosed in 2020, as shown in the report of global cancer statistics [1]. According to the tumor-node-metastasis classification system that doctors use to determine the status and treatment of CRC, the disease evolves in several stages. Given the increase in the global incidence of CRC and the poor 5-year survival rates for stages III and IV, determining a new treatment strategy is crucial. The Wnt signaling pathway is most frequently mutated in CRC, which increases the pathway activity. The Wnt/β-catenin signaling pathway controls vital biological processes, such as embryonic development, cell fate determination, and cell proliferation [2]. However, abnormal components or proteins in the Wnt/β-catenin pathway may cause the accumulation of β-catenin, even in the absence of Wnt ligands, leading to carcinogenesis [2,3]. The adenomatous polyposis coli (APC) protein has a high probability of mutation and was identified in 70–90% of patients with CRC [4]. An APC mutation leads to chromosomal instability that causes other mutations, such as RSK or a *p53* gene mutation, that leads to CRC [4,5].

Usually, β-catenin is a key protein that controls the activation of the Wnt/β-catenin signaling pathway. The stability of β-catenin is controlled by the destruction complex, which is composed of Axin, APC, glycogen synthase kinase 3β (GSK3β), and casein kinase 1 (CK1). In the absence of Wnt ligands, β-catenin is phosphorylated through the destruction complex, followed by ubiquitination and degradation. When the Wnt/β-catenin signaling pathway is activated by Wnt ligands, the coreceptors’ low-density lipoprotein 5/6 (LRP5/6) is phosphorylated by Dishevelled (Dvl). Phosphorylated LRP5/6 then recruits Axin and destabilizes the destruction complex, leading to the accumulation of β-catenin, which enters the nucleus to trigger the downstream signal pathway [6]. However, loss of function of the APC also causes uncontrollable transcription of the downstream genes through the excess accumulation of β-catenin and aberrant proliferation [7]. Tankyrase proteins (TNKS-1 and TNKS-2), members of the poly (ADP-ribose) polymerase enzyme (PARP) family, use NAD^+^ as the substrate for ADP-ribose and transfer ADP-ribose into the target protein (PARsylation) [6,8]. TNKSs are involved in various biological functions such as telomere maintenance, DNA repair, mitosis, and GLUT4 vesicle trafficking [9]. Additionally, TNKSs regulate the Wnt/β-catenin pathway through the PARsylation and destabilization of Axin. Because Axin is the concentration-limiting component of the destruction complex, its abundance regulates the stability of β-catenin [6]. Although many TNKS inhibitors have been identified, none of them have entered the market [10]. Hence, providing a novel TNKS inhibitor strategy may be beneficial for combating related cancers. Studies have demonstrated that inhibiting the Wnt signaling pathway through a TNKS inhibitor could potentially treat CRC [11,12].

Virtual screening (VS) is a useful technique for identifying drugs by screening small-compound databases to determine if active inhibitors or novel scaffolds, which have been applied in many studies [13,14,15,16]. Structure-based VS calculates the binding free energy or considers the features or shape of the ligand binding site. Generally, docking, structure-based pharmacophore, and molecular dynamics (MD) simulations are the approaches used in structure-based VS For a structure-based pharmacophore, the researcher builds a pharmacophore model based on the shape or features of a protein for database screening; this approach has been successfully utilized in several studies [13,17]. In general, the VS hit rates and high throughput screening rates are 1–40% and 0.01–0.14%, respectively. The common activity cutoff in VS is 1–25 μM, and using a value of less than 1 μM is rare [18]. Although VS can identify potential compounds and decrease the cost of experiments, determining the affinity of compounds of less than 10 μM still presents a challenge.

In this study we combined several methods, such as pharmacophore search and molecular docking, binding free energy calculations, and ligand interactions, to screen for new TNKS inhibitors. Our results revealed that NSC319963 has the most therapeutic potential as a novel scaffold for TNKS inhibition because it inhibits the Wnt/β-catenin signaling pathway and cell proliferation in various CRC cell lines with diverse APC genotypes. Moreover, NSC319963 can enhance the effect of 5-fluorouracil (5-FU) on CRC cells. NSC319963 can reduce the viability of CRC and may provide a new therapeutic treatment for CRC.

## 2. Materials and Methods

### 2.1. Data Set Preparation

All TNKS crystal structures were obtained from the RCSB Protein Data Bank. The structures of the ChEMBL compounds and TNKS-1 and TNKS-2 inhibitors were downloaded from the ChEMBL database, and FDA-approved drugs were acquired from DrugBank. National Cancer Institute (NCI) compounds were obtained from the National Institutes of Health public database. All compounds were prepared *in silico* by the Molecular Operating Environment software package (MOE2019.01) (http://www.chemcomp.com, accessed on 28 December 2021). The drug-like properties of the compounds were calculated using the “Filter by Lipinski and Veber Rules” module of BIOVIA Discovery Studio software package (DS2018) (https://www.3ds.com/products-services/biovia/products/molecular-modeling-simulation/biovia-discovery-studio/, accessed on 28 December 2021). Compounds were removed if they violated more than one Lipinski or Veber rule. The blood-brain barrier (BBB) permeability of the compounds was calculated through the “ADMET Descriptors” module of DS2018. The compounds were removed if the value of log(BBB) of the compound was less than −0.52. After these compounds were prepared, the conformers were generated by using the “Conformational Search” module of the MOE software. The “Stochastic” search method was selected with an RMS gradient of 0.1, a rejection limit of 100, a conformation limit of 250, and an iteration limit of 250. All conformers were used for testing or VS.

### 2.2. Pharmacophore-Based VS

To analyze the interaction patterns between ligands and TNKS, 10 TNKS structures were selected (Table S1) using the “Analyze Ligand poses” module of DS2018. Critical residue was delineated if more than five of the interactions were between the bound ligand and the residues of the TNKS structures. The pharmacophore model was generated if more than five ligand features interacted with the critical residues of the TNKS structures. Five pharmacophores were generated by MOE2019.01, in which F1 was the donor feature, F2 represented the acceptor feature, F3 stood for the aromatic feature, and F4 and F5 represented the hydrophobic and aromatic features, respectively. F2 and F3 were deemed essential because they can be found in all TNKS structures. The “PLIF” panel and “Querygenerator” tools of MOE2019.01 were executed to construct excluded volumes by using the average shape of the ligand binding sites from 10 TNKS structures. The pharmacophores and excluded volumes were utilized to test or screen compounds by using the “pharmacophore search” module in MOE2019.01. Compounds were removed if they overlapped with the excluded volumes or fit fewer than four pharmacophores (two essential pharmacophores and two other pharmacophores).

### 2.3. Structure-Based VS

After the pharmacophore search, pharmacophore docking was performed using MOE software. The TNKS-1 crystal structure (PDB: 4U6A) was selected for docking with small ligands. If the docking could fit more than four pharmacophores, the docking result was retained. To increase the VS hit rate, the binding energy was calculated using the “Calculate Binding Energies” module in DS2018 following the pharmacophore docking. The compounds were reranked on the basis of the weighted binding energies. The weighted binding energy was defined as ∆G × N_R_, where ∆G is the calculated binding energy, and N_R_ is the number of critical residues calculated with the “Analyze Ligand poses” module in DS2018.

### 2.4. MD Simulations

The ligands were treated with an antechamber tool of the AMBER program with a GAFF force field. Restrained electrostatic potential charges were calculated through Gaussian 09 using Hartree−Fock (HF)/6-31G(d). Short-term MD simulations were performed using the GROMACS 2018 program with Amber99 SB-ILDN force field. The ligand-bound TNKS structure was placed in a cubic TIP3P water box (8.2 × 8.2 × 8.2 nm^3^) and then neutralized with ions (Na^+^ and Cl^−^) to generate a 0.15 mol/L NaCl solution. A steepest decent energy minimization was performed in 2000 steps. After energy minimization, a 1000 ps isobaric-isothermic (NPT) ensemble was performed. Long-range electrostatic interactions were calculated using the particle mesh Ewald method with grid dimensions of 0.12 nm and an interpolation order of 4. The short-range nonbonded interactions were treated with a cutoff radius of 1.0 nm, and van der Waals potentials were switched and started at 0.8 nm. The velocity-rescaling thermostat was set to a constant temperature (310 K) and pressure (1 bar). Temperatures of the complexes and solvents were separately coupled with a coupling time of 0.1 ps. Isotropic pressure coupling was applied with a coupling time of 0.1 ps and compressibility of 4.5 × 10^−5^ bar^−1^ in the x-, y-, and z-directions. After equilibration, the MD simulations were conducted without restraint for 10 ns. The MM/PBSA binding energies of the TNKS inhibitors were calculated using the GROMACS program tool g_mmpbsa [19]. More detailed settings of the free energy calculations were described in our previous studies [20,21,22].

### 2.5. Compound Similarity

The structures of NSC319963 and NSC295092 were calculated to verify the novelty of the scaffold structure. The 795 TNKS inhibitors were selected with the “Find Similar Molecules by Fingerprints” module in DS2018. NSC319963 was defined as a reference structure. The fingerprint ECFP_4 was selected, and the cutoff was set to 0.4 [23,24].

### 2.6. The Source of Compounds

The XAV939 compound was purchased from Selleckchem (Sylvanfield Drive, Houston, TX, USA), and the 5-FU was obtained from Medchemexpress (Monmouth Junction, NJ, USA). The NCI compounds were acquired from the National Institute of Health (Bethesda, MD, USA). The XAV939 and NCI compounds were dissolved in dimethyl sulfoxide (DMSO), and the 5-FU was dissolved in ddH_2_O. All the compounds were stored at −20 °C.

### 2.7. Cell Culture

The DLD-1 cells were maintained in RPMI 1640 (Gibco/Invitrogen, Carlsbad, CA, USA), supplemented with 10% heat-inactivated fetal bovine serum (FBS; Sigma-Aldrich, St. Louis, MO, USA), 2 mM L-Glutamine, 4.5 g/L glucose, 10 mM HEPES, 1 mM sodium pyruvate, and penicillin-streptomycin at 37 °C with 5% CO_2_. The SW403 cells were maintained in Leibovitz’s L-15 Medium with 10% FBS and penicillin-streptomycin at 37 °C. The HCT-116 cells were maintained in McCoy’s 5A Medium (Corning, Glendale, AZ, USA), 10% FBS, and penicillin-streptomycin at 37 °C with 5% CO_2_. All human colon cancer cell lines were obtained from the Bioresource Collection and Research Center (Food Industry Research and Development Institute, Hsinchu, Taiwan).

### 2.8. TNKS-1 Activity Assay

All the NCI compounds were screened with a TNKS-1 histone ribosylation colorimetric assay kit (BPS Bioscience, San Diego, CA, USA), which tested their ability to inhibit TNKS-1 activity. The plate was coated overnight with histone, and 150 μL of a blocking buffer was added over 60 min at room temperature. Then, the reaction buffer was prepared at room temperature with various concentrations of compounds, and 30 ng/well of TNKS-1 was added. A total of 50 μL of streptavidin-HRP was then added at room temperature for 30 min. Finally, 100 μL Colorimetric HRP Substrate was added for 20 min at room temperature, and 100 μL of 2 M sulfuric acid was added to end the reaction. Absorbance was detected at 450 nm using a Varioskan LUX Multimode Microplate Reader (Thermo Fisher Scientific, Waltham, MA, USA). The inhibition percentage was calculated using the following equation:

Inhibition rate = 1 − (sample − blank control)/(negative control − blank control) × 100%(1)

where the blank control was the absent compound plus TNKS-1. The negative control was TNKS-1 only.

### 2.9. SuperTopFlash Reporter Assay

A total of 30,000 DLD-1 cells were seeded on a 24-well plate overnight. The cells were transfected with a 20:1 ratio of a M50 Super 8 × TOPFlash Firefly luciferase reporter gene (Addgene, Watertown, MA, USA) and pGL4.74 [hRluc/TK] Renilla luciferase reporter gene (Promega, Madison, WI, USA) using ViaFect Transfection Reagent (Promega, Watertown, MA, USA) for 24 h. The various concentrations of compounds were treated for 24 h, and the cells were lysed and analyzed with a dual-luciferase reporter assay system (Promega, Watertown, MA, USA). Luminescence was analyzed using a Varioskan LUX Multimode Microplate Reader. The luciferase reporter activity was normalized with respect to Renilla luciferase activity and expressed as a percentage of the control activity.

### 2.10. Western Blot

A total of 1 million SW403 cells were seeded on 6-cm dishes overnight, and various compounds were treated for 48 h. The cell lysates were collected, and samples were loaded onto sodium dodecyl sulfate-polyacrylamide gel through electrophoresis and transferred onto polyvinylidene difluoride blotting membranes. The membranes were blocked using 5% skim milk with phosphate-buffered saline (PBST) for 1 h. An anti-Axin2 antibody (Cell Signaling Technology, Danvers, MA, USA) was added overnight in 4 °C conditions. The membrane was washed three times with PBST, and either an anti-active β-catenin antibody (Millipore, Burlington, MA, USA), anti-total β-catenin antibody (Genetex, Irvine, CA, USA), or anti-α-tubulin antibody (Genetex, Irvine, CA, USA) was added for 1 h. The membrane was then washed three times with PBST. An ECL reagent was added, and the luminescence was analyzed.

### 2.11. Colony Formation Assay

A total of 2000 or 100,000 cells/well of DLD-1, HCT-116, and SW403 were seeded overnight in 6-well plates. The compounds were treated the following day, and the medium with the compound was replenished every 3 days for 12 or 18 days. Colonies were stained with 2 mg/mL crystal violet in methanol for 30 min. The colonies were dissolved with 15% acetic acid, and absorbance was analyzed at 540 nm using the Varioskan LUX Multimode Microplate Reader.

### 2.12. Statistical Analysis

All experiments were repeated at least three independent times, except for the TNKS-1 activity assay, which was repeated once or twice. Data were presented as the mean ± standard deviation (SD). The pIC_50_ response was measured using nonlinear curve fitting with the dose–response module in OriginPro 2020, and the pIC_50_ was calculated using the equation −log_10_(IC_50_). The significance of the various concentration groups was analyzed with a one-way analysis of variance with Tukey’s multiple comparisons test module in OriginPro 2020. Statistical significance was set at *p* < 0.05 and is indicated with an asterisk.

## 3. Results

### 3.1. Structure-Based Pharmacophore VS

**Figure 1:**
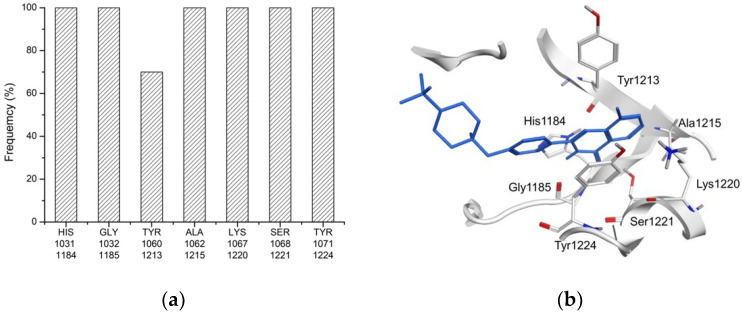
The critical residues of ligand binding sites on TNKS-1 and TNKS-2. (**a**) The seven critical residues determined of TNKS-1 and TNKS-2 are His1184, His1031, Gly1185, Gly1032, Tyr1213, Tyr1060, Ala1215, Ala1062, Lys1220, Lys1067, Ser1221, Ser1068, Tyr1224, Tyr1071, respectively. (**b**) Co-crystal ligand of TNKS-1 (4U6A) interact with the seven critical residues.

**Figure 2:**
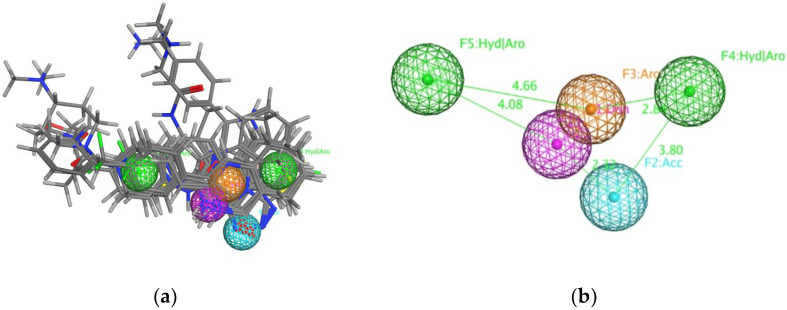
The defined pharmacophore model of TNKS protein. (**a**) 10 crystal structures and co-crystal ligands were superposed. (**b**) The purple is F1 donor, the cyan is F2 acceptor, the orange is F3 aromatic, and the two greens are F4 and F5 aromatic or hydrophobic. The F2 and F3 are defined as essential ligand features.

In the VS model training, the structure-based pharmacophore search was initially performed to identify potential TNKS inhibitors. A total of 10 TNKS crystal structures (Table S1) were selected to identify critical residues in TNKSs. Seven critical residues interacted with the cocrystal ligand in TNKSs, namely His1184 (TNKS-1) and His1031 (TNKS-2), Gly1185 (TNKS-1) and Gly1032 (TNKS-2), Tyr1213 (TNKS-1) and Tyr1060 (TNKS-2), Ala1215 (TNKS-1) and Ala1062 (TNKS-2), Lys1220 (TNKS-1) and Lys1067 (TNKS-2), Ser1221 (TNKS-1) and Ser1068 (TNKS-2), and Tyr1224 (TNKS-1) and Tyr1071 (TNKS-2; Figure 1 and Figure S1). The five pharmacophores (F1–F5 in Figure 2 and Table 1) were generated on the basis of critical residues and cocrystal ligand interactions, and F2 and F3 were deemed essential pharmacophores because their features were present in all of the TNKS crystal structures. A total of 795 TNKS inhibitors, 1945 FDA-approved compounds, and 6618 ChEMBL compounds were pooled together for the pharmacophore search. The results identified 357 TNKS inhibitors, 160 FDA-approved compounds, and 937 ChEMBL compounds. The hit rate of the TNKS inhibitors in the total compounds increased from 8.5% [795/(795 + 1945 + 6618)] to 24.55% [357/(357 + 160 + 937)]. Thus, the compounds filtered through the pharmacophore search could increase the hit rate of TNKS inhibitors in the databases.

**Figure 3:**
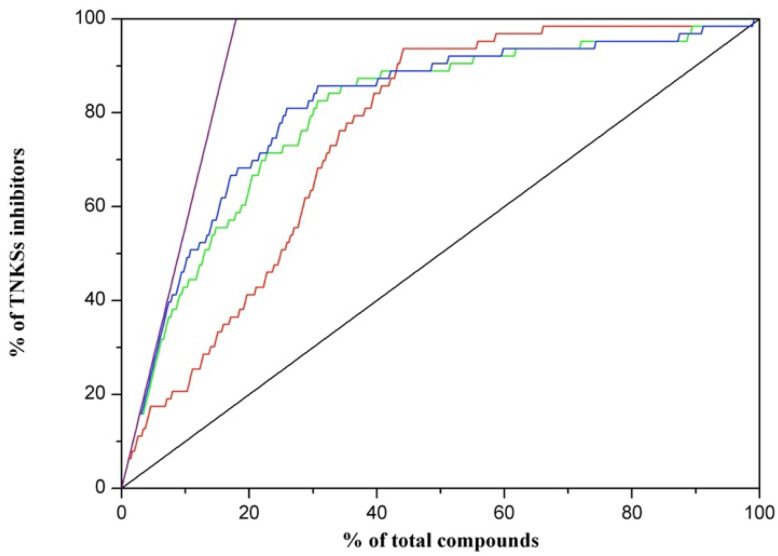
The validation of different methods for virtual screening. The black line is random module. The purple line is ideal module. The red line is pharmacophore docking module. The green line is the module of binding energy calculation from pharmacophore docked structure. The blue line is the module of weighted binding energy calculation from pharmacophore docked structure.

The combination of docking and a pharmacophore-based search was recommended to increase the hit rate, compared with using only one approach [25]. The 50 TNKS-1 inhibitors, 50 TNKS-2 inhibitors with an IC_50_ value of less than 2 μM, 160 FDA-approved compounds, and 937 ChEMBL compounds that were hit in the pharmacophore search were then selected for pharmacophore docking. The results indicated that 33 TNKS-1, 30 TNKS-2 inhibitors, 29 FDA-approved compounds, and 259 ChEMBL compounds were hit. We ranked these compounds on the basis of their docking scores, and we analyzed the true positive hit rates, as presented in Figure 3 and Table 2. The pharmacophore docking indicated that 17.46% of the TNKS inhibitors were present in the top 5% of the ranked compounds, the random ranking was 5.13%, and the ideal ranking was 28.57%. We redocked the cocrystal ligands of the TNKSs to illustrate that except for 3UH2 with a high RMSD value of 3.39 Å and 4BJC, for which the pharmacophore docking could not fit the pharmacophore, the cocrystal structures had low RMSD values (Figure S2). The hit rate of the TNKS inhibitors for pharmacophore docking based on docking scores was more accurate than random ranking to illustrate that the cocrystal ligands of TNKSs have low RMSD values.

### 3.2. Binding Free Energy Calculation Applied to Improve Correlation Coefficient

The calculation of MM/PBSA binding free energy from the minimized structures could result in a satisfactory correlation coefficient between affinity and binding free energy [26,27]. Therefore, the binding energy of compounds was calculated and ranked with the “Calculate Binding Energies” module in DS2018. The results indicated that the hit rate of the TNKS inhibitors was 25.4% in the top 5% compounds of all the ranking compounds, meaning that the hit rate when calculating binding energy is more accurate than that obtained through pharmacophore docking only. Moreover, studies have also demonstrated that the combination of docking scores and residue interactions can increase the VS hit rate [15,28]. Hence, we combined the binding energy and critical residues methods to rank the compounds by their weighted binding energy scores (Figure 3 and Table 2). The hit rate of the TNKS inhibitors increased to 26.98% in compounds ranking in the top 5%. The hit rate of the weighted binding energy was more accurate than that of the DS2018 binding energy calculations in this study.

**Figure 4:**
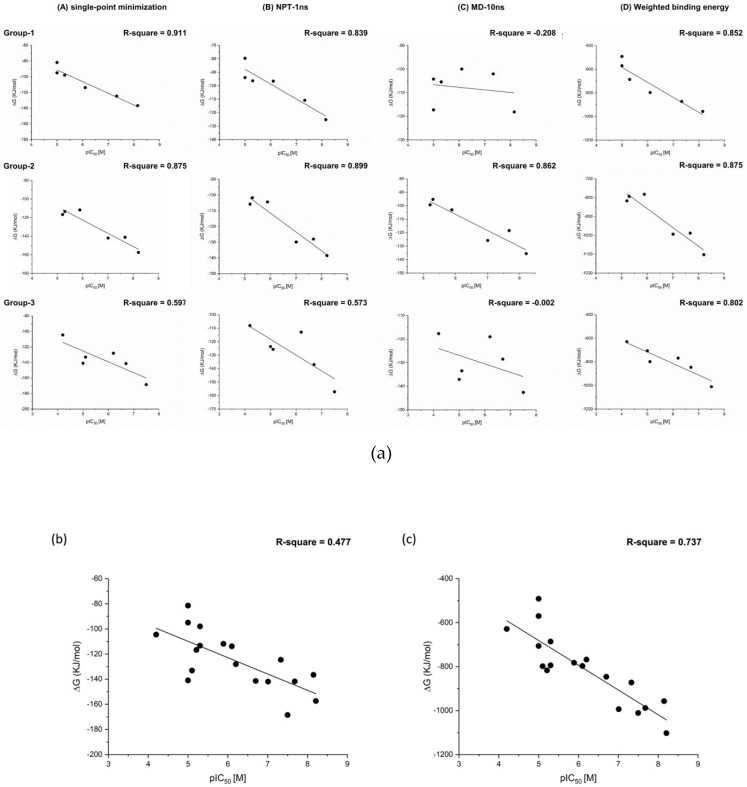
The relationships between the experimental pIC_50_ of TNKS-1 inhibitors and binding free energy (kJ/mol) calculations (**a**) column A from single-point minimization; column B from NPT-based simulation; column C from MD-based simulation; column D from weighted binding energy calculation. (**b**,**c**) The relationship of all experimental pIC_50_ of TNKS-1 inhibitors and binding free energy. (**b**) from single-point minimization (**c**) from weighted binding energy calculation.

Other than the binding energy calculated with the DS2018 software, the MM/PBSA binding free energy calculations from the GROMACS software were also performed for comparison. Three scaffold groups of TNKS inhibitors and their derivatives were selected to assess the MM/PBSA calculations (Table S2). All the docking modes were calculated at minimization, 1 ns NPT ensemble simulation, and 10 ns MD simulation. The results demonstrated that minimization and the NPT ensemble had suitable correlation coefficients between the pIC_50_ and binding energy calculated in scaffold groups 1 and 2, but had a poor correlation coefficient for group 3 (Figure 4a). However, the rescored binding free energies of all pooled scaffold groups of TNKS inhibitors and their derivatives determined through minimization revealed a worse correlation coefficient compared with the rescored weighted binding energy of all the scaffold groups of TNKS inhibitors (Figure 4b,c). Hence, the combination of binding energies and critical residues improved the correlation coefficient for the pIC_50_ in this study.

### 3.3. The Compositive VS Model Applied for New Scaffold Screening

**Figure 5:**
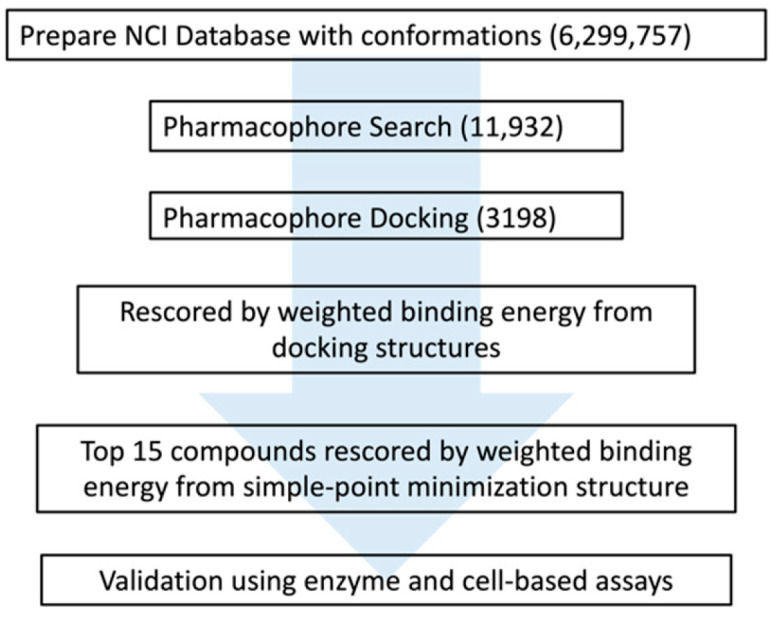
The workflow of proposed combinatorial virtual screening in this study. From NCI database, 6,299,757 conformations were generated. A total 11,932 compounds were hit after pharmacophore search and 3198 compounds were hit by the followed pharmacophore docking. These compounds were rescored by the weighted binding free energy calculation and top 15 compounds were selected for further validation by using enzyme and cell-based assays. The blue arrow just meant the progress direction of the workflow (from top to down).

The NCI database, which contains information on approximately 260,000 compounds, was selected for VS in this study. The drug-likeness of the compound was calculated with Lipinski’s rules and Veber’s rules. Although the incidence of brain metastasis from CRC is low [11], the global incidence of CRC is expected to increase [18]. Some patients have been diagnosed with brain metastasis from CRC [29]. If the compound could penetrate the BBB, it may be able to resist CRC-induced brain metastasis. Therefore, the compounds were filtered according to BBB permeability in DS2018. After the database was filtered by Lipinski’s rules, Veber’s rules, and BBB permeability, approximately 130,000 compounds remained, which generated approximately 6,300,000 conformers using MOE2019. Next, the database was screened according to the proposed workflow (Figure 5). Finally, the top 15 compounds were selected and ranked according to their calculated binding energy scores from the minimized structures (Table 3).

### 3.4. Inhibition of TNKS-1 and Wnt Signaling Test

TNKS inhibitors catalyze NAD^+^ to generate ADP-ribose polymers on target proteins [31]. Therefore, a TNKS-1 histone ribosylation colorimetric assay was used to evaluate the inhibition efficiency of the compounds, and 10 μM of each compound was used for screening. For the TNKS-1 enzyme-based assay, five compounds (NSC319963, NSC315247, NSC295092, NSC123012, and NSC102371) demonstrated >50% TNKS-1 inhibition (Table 4). Although the weighted binding energy (minimization-based) demonstrated a high correlation coefficient, the hit rate confirmed with the enzyme-based assay was only 45.5%. TNKSs control Wnt/β-catenin signaling through the stabilization of the β-catenin destruction complex. Inhibition of TNKS activities increases β-catenin through the decrease of the β-catenin destruction complex in the cytoplasm [6]. The active β-catenin associates with T-cell factor/lymphoid enhancer factor (TCF/LEF) transcription factors and triggers downstream signal transduction [32]. A SuperTopFlash reporter has seven TCF/LEF regions that can amplify the signal of β-catenin [33]. Thus, the five compounds were screened using a SuperTopFlash reporter assay at 10 μM in DLD-1 cells with constitutively active β-catenin [34]. NSC295092 and NSC319963 demonstrated a Wnt activity inhibition of >50% (Table 4). NSC102371, NSC123012, and NSC315247 demonstrated suitable TNKS-1 inhibition effects at 10 μM, but the Wnt activity inhibition effect was less than 50%. The IC_50_ of NSC295092 and NSC319963 was further evaluated, and the values of TNKS-1 inhibition were 7.18 and 7.66 nM, respectively. The IC_50_ of the Wnt activity inhibition of NSC295092 and NSC319963 was 5.11 and 5.45 nM, respectively.

**Figure 6:**
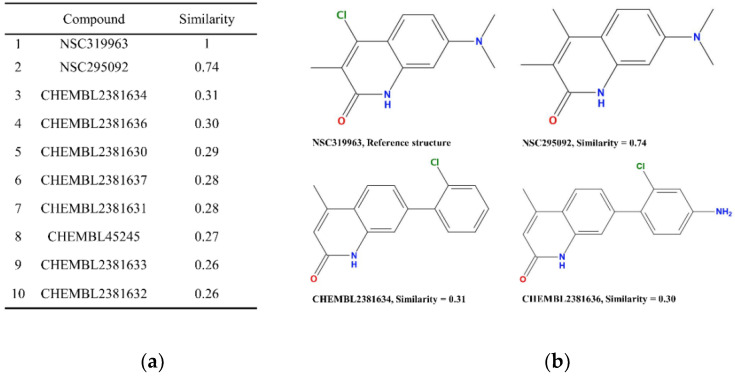
The similarity of screened compounds NSC319963 and NSC295092. (**a**) The structure similarity of NSC319963, NSC295092 and know TNKSs inhibitors. (**b**) The most similar compounds and their structures.

**Figure 7:**
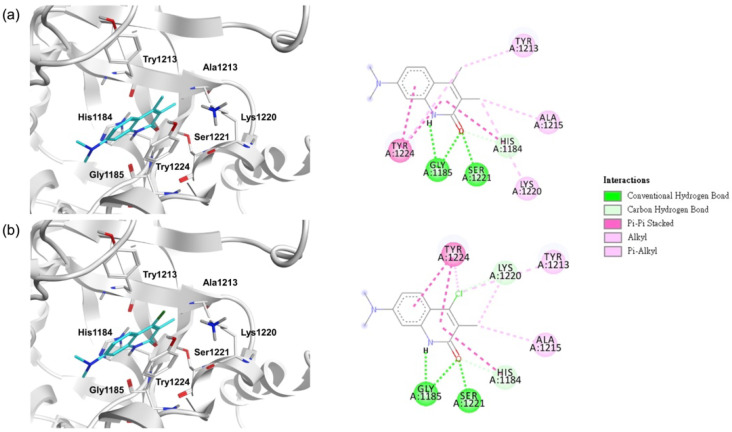
The docking modes of NSC319963 and NSC295092. (**a**) Docking model of NSC295092 and 2D interaction map. (**b**) Docking model of NSC319963 and 2D interaction map.

TNKS inhibitors have decreased β-catenin protein expression with stable Axin [12]. Therefore, we evaluated the protein expression of active β-catenin, total β-catenin, and Axin2 through Western blot. We determined that NSC295092 and NSC319963 can decrease active β-catenin and total β-catenin by promoting Axin2 in SW403 (Figure S3). To determine whether NSC295092 and NSC319963 were novel scaffolds of TNKS inhibition, a two-dimensional fingerprints method (ECFP_4) was performed with DS2018 software, and NSC319963 was set as the reference structure to compare with 795 known TNKS inhibitors and NSC295092. If the Tanimoto coefficient was more than 0.4, the compound had a similar scaffold as the reference compound [23,24]. The Tanimoto coefficient of NSC295092 was 0.74, and the highest value of the other known TNKS inhibitors was 0.31 (Figure 6). Therefore, NSC319963 and NSC295092 had a similar scaffold and may be considered novel TNKS inhibitors. The docking modes of NSC295092 and NSC319963 revealed similar interactions with TNKS-1 (Figure 7). In all of the compounds, His1184 and Try1224 formed π-alkyl with 1H-quinolin-2-one ring, Gly1185 and Ser1221 formed hydrogen bonds with 1H-quinolin-2-one ring, and Ala1215 and Lys1220 formed alkyl with the 3-methyl group. However, Tyr1213 and Tyr1224 formed alkyl with the 4-methyl group only in NSC295092, and Tyr1213 and Tyr1224 formed alkyl with the 4-chloro group only in NSC319963. Lys1220 formed a carbon hydrogen bond with the 4-chloro group in NSC319963. The docking modes of compounds NSC295092 and NSC319963 with TNKS-1 were also superposed with compound XAV939 showing these compounds are in the same binding pocket of TNKS-1 and with similar interactions (Figure S4).

### 3.5. NSC319963 Inhibits Cell Growth on Colony Formation in CRC Cell Lines

**Figure 8:**
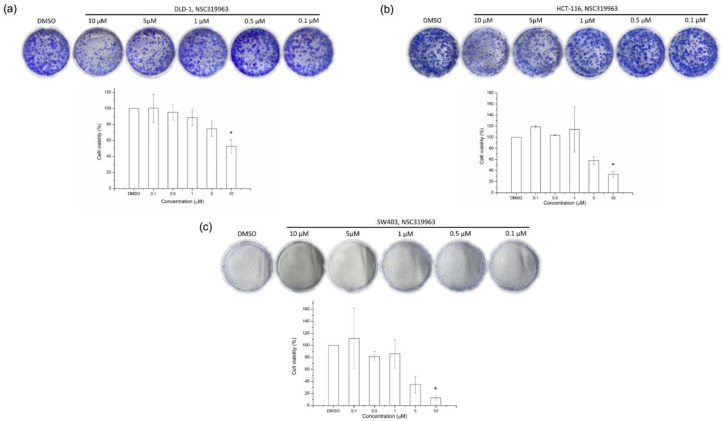
Inhibition of cell growth in APC colorectal cancer cells. (**a**) DLD-1 (**b**) HCT-116 and (**c**) SW403 cells were incubated with various concentrations of NSC319963 for 10 to 12 days. Colony formation was assessed to detect the effect of NSC319963 on cell growth. Colony formation was counted and presented as bar graphs. Each column represents the mean ± SD with triplicate experiments. * *p* < 0.05, compared to DMSO group.

Aberrant Wnt signaling, which normally originates from an APC mutation, was observed in 70–90% of patients with CRC [4]. The short-form APC mutation of the CRC cells was sensitive to TNKS inhibitors. The NSC319963 had a superior pIC_50_ for Wnt inhibition than NSC295092. Therefore, the CRC cell lines, namely SW403 with short-form APC mutation, DLD-1 with APC mutation, and HCT-116 with wild-type APC, were selected to evaluate the inhibition of colony formation of NSC319963 for 18 days. NSC319963 inhibited colony formation in all CRC cell lines at 10 μM, which indicates that NSC319963 can inhibit SW403, DLD-1, and HCT-116 cell growth. (Figure 8).

## 4. Discussion

In this study, we conducted a more efficient VS of new scaffolds of TNKS inhibitors compared with traditional screening methods after first completing screening model training. Simple docking methods, such as rigid body or induced fit docking, have been used in many studies [15,35,36] and may easily be biased because of their oversimplified simulation systems (Figure S2). The pharmacophore method is used to identify potential compounds through pharmacophores, which are built with ligand or protein features, and the screened compounds are deemed potential compounds if they can fit the pharmacophores. Although this method also successfully identified some protein inhibitors, the results contained many false positives [13,17]. Studies have used the pharmacophore and docking methods to determine TNKS inhibitors with suitable affinity in an enzyme-based assay [37] and have indicated that the combination of methods can increase the hit rate compared with a single approach [25,36]. Therefore, the combination of pharmacophore and docking methods provides the benefits of both methods and can increase the VS hit rate or affinity. According to our model training results, the combination increased the VS hit rate by decreasing the incidence of bias in the docking model (Figure 3 and Table 2).

Studies have demonstrated that calculating the binding free energy from the structure after minimization or MD simulations by using the MM/PBSA method can result in a suitable correlation coefficient between the affinity and binding free energy [26,27]. Therefore, we calculated the binding free energy from the pharmacophore docking method in this study. The results (Figure 3 and Table 2) indicated that calculating binding free energy on the basis of the docking structure through MM/PBSA could increase the VS hit rate from 17.46% to 25.40%. Moreover, given that MM/PBSA calculations could yield more accurate results when combined with residue interactions than without residue interactions, the VS hit rate could be increased from 25.40% to 26.98%, which is consistent with the results of a previous study [15]. After the screening model training, a revised model was applied to search the NCI database for the new scaffolds of TNKS inhibitors. This method led to five compounds with over 50% inhibition, according to the TNKS-1 enzyme-based assay (5/11), and a hit rate of 18.2% (2/11) in the final cell assay, in which two compounds were hit. Although the hit rate was poor, the potency of the hit compounds was higher than in some studies [14,17,35].

The docking model of NSC319963 and NSC295092 (Figure 7) demonstrated that both compounds interacted with seven critical residues and were derivatives. The MM/PBSA values obtained from the minimized structures of NSC319963 and NSC295092 were −875.90 and −852.57 kJ/mL, respectively. Hence, NSC319963 had stronger affinity than NSC295092. NSC319963 demonstrated the greatest potential as a TNKS inhibitor according to the TCF luciferase reporter assay (Table 3), which was consistent with the MM/PBSA values from the minimized structure. Calculations of binding free energy from the minimized structures through MM/PBSA may be conducted to modify the structure, but they are unsuitable for VS because of the computing cost. Docking models of NSC319963 and NSC295092 provided molecular insights into the compounds that inhibit TNKS activity. Therefore, a combination of the docking model and the calculation of binding free energy from the minimized structures through MM/PBSA can optimize NSC319963 to increase the compound affinity.

**Figure 9:**
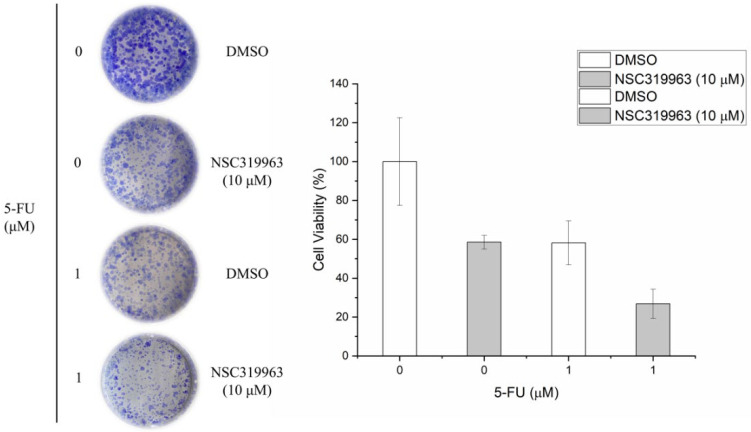
NSC319963 enhances the chemosensitivity of 5-FU in DLD-1 cells. DLD-1 cells were treated with different concentrations of NSC319963 and 5-FU for 12 days. Colony formation was counted and presented as bar graphs. Each column represents the mean ± SD with triplicate experiments.

To further verify the inhibitory effect of NSC319963 on CRC, three cell lines, namely SW403, DLD-1, and HCT-116, were assessed for colony formation. As presented in Figure 8, 10 μM NSC319963 produced greater inhibitory effects of cell viability on the SW403 (less than 20%) than the HCT-116 (less than 40%) and DLD-1 (50%) cell lines. The possibility of SW403 containing a short-form APC mutation and DLD-1 and HCT-116 containing an APC mutation and wild-type APC, respectively, may indicate that the regulatory effect of NSC319963 on Axin was associated with the activity of APC. Because APC may be mutant in DLD-1 cells, the interaction between Axin and APC might decrease. The effect of NSC319963 on β-catenin accumulation might also decrease, which may lead to a poor effect on colony formation. By contrast, SW403 contains a short-form APC mutation; therefore, the region of the Axin/APC interaction remains normal. The short-form APC mutation may increase the possibility of an Axin/APC interaction; therefore, NSC319963 has a greater inhibitory effect on SW403 cells than on DLD-1 cells. However, NSC319963 also demonstrated a synergistic effect with 5-FU on DLD-1 viability (Figure 9), which indicates that NSC319963 can enhance the chemosensitivity of 5-FU on CRC.

## 5. Conclusions

In conclusion, the Wnt signaling pathway is the most common mutation in CRC, and an abnormal Wnt signaling pathway increases cancer cell growth, invasion, and resistance to treatment [38,39,40]. TNKS inhibitors have the potential to treat CRC by decreasing Wnt signaling pathway activity [11,12]. In this study, an improved VS strategy was proposed for screening potential BBB-permeable TNKS inhibitors. This strategy combined pharmacophore, docking, MM/PBSA, and critical residue interactions to determine a novel scaffold for TNKS inhibition from the NCI database. The NSC319963 compound inhibits TNKS and Wnt signaling pathway activities, decreases SW403 cells growth, and is nontoxic to HMEC-1 cells. According to the Tanimoto coefficient, NSC319963 is a novel scaffold for TNKS inhibition. Thus, our VS strategy successfully identified a novel scaffold for TNKS inhibition with suitable inhibition of the Wnt signaling pathway ability (pIC_50_ = 5.59). This strategy can also be applied in future studies to identify other protein inhibitors.

## Figures and Tables

**Table 1:** List of pharmacophore model.

Pharmacophore		Frequency
F1	Donor	7/10
F2	Acceptor	10/10 ^a^
F3	Aromatic	10/10 ^a^
F4	Aromatic or Hydrophobic	9/10
F5	Aromatic or Hydrophobic	6/10
Search hit, at least 4 hits, 2 Essential + 2 Others		

^a^ defined as an essential pharmacophore.

**Table 2:** Hit rates of different docking methods used for virtual screening.

Top (%)	Random (%)	Docking (%)	Binding Energy (%)	Weighted Binding Energy (Docking-Based) (%)	Ideal (%)
1.00	1.14	6.35	6.35	6.35	6.35
5.00	5.13	17.46	25.40	26.98	28.57
10.00	10.23	20.63	42.86	49.21	57.14
15.00	15.10	33.33	55.56	58.73	84.12
18.00	17.95	36.51	58.73	66.67	100.00

**Table 3:** Top 15 compounds selected in the combinatorial virtual screening.

	NSC ID	Predicted pIC_50_^a^	Weighted Binding Energy (Docking-Based) ^b^	Weighted Binding Energy (Minimization-Based) ^c^
1	123012	6.23	−333.97	−819.40
2	295092	6.52	−321.65	−852.57
3	401309	6.53	−313.99	−853.66
4	158478	6.27	−312.32	−823.95
5	12375	5.52	−307.31	−739.31
6	345683	5.57	−304.95	−745.41
7	188041	7.03	−303.33	−909.31
8	400085	6.26	−301.18	−823.19
9	319963	6.73	−300.46	−875.90
10	102371	6.99	−299.06	−905.23
11	315247	7.07	−298.22	−914.52
12	670437	9.06	−295.86	−1138.15
13	670428	9.18	−295.52	−1151.59
14	121291	4.58	−289.87	−633.33
15	102045 ^d^	6.63	−289.48	−864.12

^a^ Predicted pIC_50_ was referred from y = −112.87x−116.23 which from minimize-based weighted binding energy; ^b,c^ The unit is kJ/mL; ^d^ The experiment pIC50 of TNKS-1 is 6.44 [30].

**Table 4:** Summary of selected compounds and XAV939 for 10 μM inhibition and pIC_50_ in TNKS-1 and TCF-reporter activity assays.

	Compound	Pred pIC_50_ ^b^	% of Inhibition at 10 μM		pIC_50_	
			TNKS-1 ^c^	TCF-Reporter	TNKS-1	TCF-Reporter
1	XAV939 ^a^		102.34	74.73 ± 2.52	8.45 ± 0.29	6.85 ± 0.06
2	NSC670437	9.06	−7.33	nd	nd	nd
3	NSC670428	9.18	5.52	nd	nd	nd
4	NSC401309	6.54	20.14	nd	nd	nd
5	NSC400085	6.54	16.29	nd	nd	nd
6	NSC319963	6.73	100.75	73.47 ± 6.02	7.66 ± 0.05	5.45 ± 0.06
7	NSC315247	7.07	60.07	−15.93 ± 28.86	nd	nd
8	NSC295092	6.53	102.09	55.33 ± 1.51	7.18 ± 0.05	5.11 ± 0.05
9	NSC188041	7.03	11.84	nd	nd	nd
10	NSC158478	6.26	−8.19	nd	nd	nd
11	NSC123012	6.24	50.92	45.23 ± 19.02	nd	nd
12	NSC102371	6.99	88.54	−23.67 ± 11.01	nd	nd

^a^ Control inhibitor; ^b^ Pred pIC_50_ was referred from y = −112.87x−116.23; ^c^ One or two independent experiments. nd: not determined.

## Data Availability

Not applicable.

## Supplementary Materials

The following are available online at https://www.mdpi.com/article/10.3390/biomedicines10010143/s1, Table S1: 10 TNKS crystal structures list, Table S2: Three group of different scaffold TNKSs inhibitors and their derivatives, Figure S1: Detailed interaction maps of 10 co-crystal ligands with the surrounding residues of their crystal structures, Figure S2: Redocking co-crystal ligand by pharmacophore docking. The cyan is the crystal structure, and purple is the docking pose, Figure S3: Cell lysates were collected at 48 hr and subjected to western blot for detection of active β-catenin, total β-catenin and AXIN2, Figure S4: The superposition of compound XAV939 with screened compounds NSC319963 and NSC295092 in the binding pocket of TNKS-1. References [11,41,42,43,44,45,46,47,48,49] are cited in the Supplementary Materials.
