# Supplementary material for: Combinatorial Virtual Screening Revealed a Novel Scaffold for TNKS Inhibition to Combat Colorectal Cancer
Source: Biomedicines. 2022 Jan 10;10(1):143. doi: 10.3390/biomedicines10010143 (PMC8773749; doi:10.3390/biomedicines10010143)

Supporting information

Combinatorial Virtual Screening Revealed a Novel Scaffold for TNKS Inhibition to Combat Colorectal Cancer

Chun-Chun Chang[1,2], Sheng-Feng Pan[3], Min-Huang Wu[2], Chun-Tse Cheng[4], Yan-Rui Su[2], Shinn-Jong Jiang[3*] and Hao-Jen Hsu[3,4*]

[1] Department of Laboratory Medicine, Hualien Tzu Chi Hospital, Buddhist Tzu Chi Medical Foundation, Hualien 97004, Taiwan
[2] Department of Laboratory Medicine and Biotechnology, College of Medicine, Tzu Chi University, Hualien 97004, Taiwan
[3] Department of Biochemistry, School of Medicine, Tzu Chi University, Hualien 97004, Taiwan
[4] Department of Life Sciences, College of Medicine, Tzu Chi University, Hualien 97004, Taiwan
* Correspondence: Jiang and H.-J. Hsu contributed equally to this study as the corresponding authors
* Correspondence: hjhsu32@mail.tcu.edu.tw (H.J.H.); sjjiang@mail.tcu.edu.tw (S.J.J.); Tel.: (+886-3-8565301 ext. 2643) (H.-J.H.)

Table S1. 10 TNKS crystal structures list.

	TNKSs	PDB Code	Compound Name	TNKS-1 Affinity (nM)	TNKS-2 Affinity (nM)	Ref
1	TNKS-1	3UH2	PJ34	1690	2900	[41,42]
2	TNKS-1	3UH4	XAV939	94.60	5.20	[41,42]
3	TNKS-1	4KRS	Compound 4	299	38	[43]
4	TNKS-1	4U6A	Compound 21	13[a]		[44]
5	TNKS-2	3MHJ	Compound 106	1410 (Kd)	3360 (Kd)	[45]
6	TNKS-2	4AVW	TIQ-A	200	24	[46]
7	TNKS-2	4BJC	Rucaparib	25	14	[46]
8	TNKS-2	4HKI	Compound 1	320	140	[47]
9	TNKS-2	5DCZ	Compound 23	7.30 (pIC_{50})	6.80 (pIC_{50})	[11]
10	TNKS-2	5ZQO	compound 1a	20.50	19.40	[48]

[a]The experiment only show IC_{50} of TNKSs.

Table S2. Three group of different scaffold TNKSs inhibitors and their derivatives.

	Group	ChEMBL ID	Exp. pIC_{50}	Binding Energy (Minimized)[b]	Weighted Binding Energy (Minimized)[c]	NPT 1 ns	MD 10 ns	Ref
1	1	2431803	8.15	−136.75	−957.22	−125.16	−118.15	[47]
2	1	16861	7.33	−124.62	−872.32	−110.78	−102.03	[47]
3	1	327209	6.10	−113.82	−796.74	−96.59	−99.95	[47]
4	1	16782	5.30	−98.03	−686.23	−96.34	−105.40	[47]
5	1	2431807	> 5.00[a]	−81.88	−491.29	−79.62	−104.21	[47]
6	1	147722	> 5.00	−94.98	−569.88	−93.95	−117.26	[47]
7	2	3589285	8.21	−157.49	−1102.44	−138.50	−135.57	[49]
8	2	3589284	7.68	−141.19	−988.33	−128.03	−118.42	[49]
9	2	3589281	7.01	−141.97	−993.79	−129.88	−125.84	[49]
10	2	3589247	5.89	−111.76	−782.33	−104.38	−103.09	[49]
11	2	3589254	5.30	−113.41	−793.86	−101.69	−95.30	[49]
12	2	3589258	5.21	−116.71	−816.98	−105.75	−99.34	[49]
13	3	4104232	7.50	−168.44	−1010.64	−157.20	−142.59	[11]
14	3	4068660	6.70	−141.05	−846.30	−136.95	−128.48	[11]
15	3	4086359	6.20	−127.96	−767.76	−112.94	−119.01	[11]

16	3	4095578	5.10	−133.02	−798.12	−125.72	−133.42	[11]
17	3	4080509	> 5.00	−141.17	−705.85	−123.63	−137.11	[11]
18	3	4105641	4.20	−104.81	−628.86	−108.07	−117.67	[11]

[a]The experimental pIC_{50} was more than 5.00 and defined as 5.00; [b]The binding energy was from steep decent minimization; [c]The weighted binding energy was from ΔG Í N_R. pIC_{50} is negative logarithm of the IC_{50} (M).

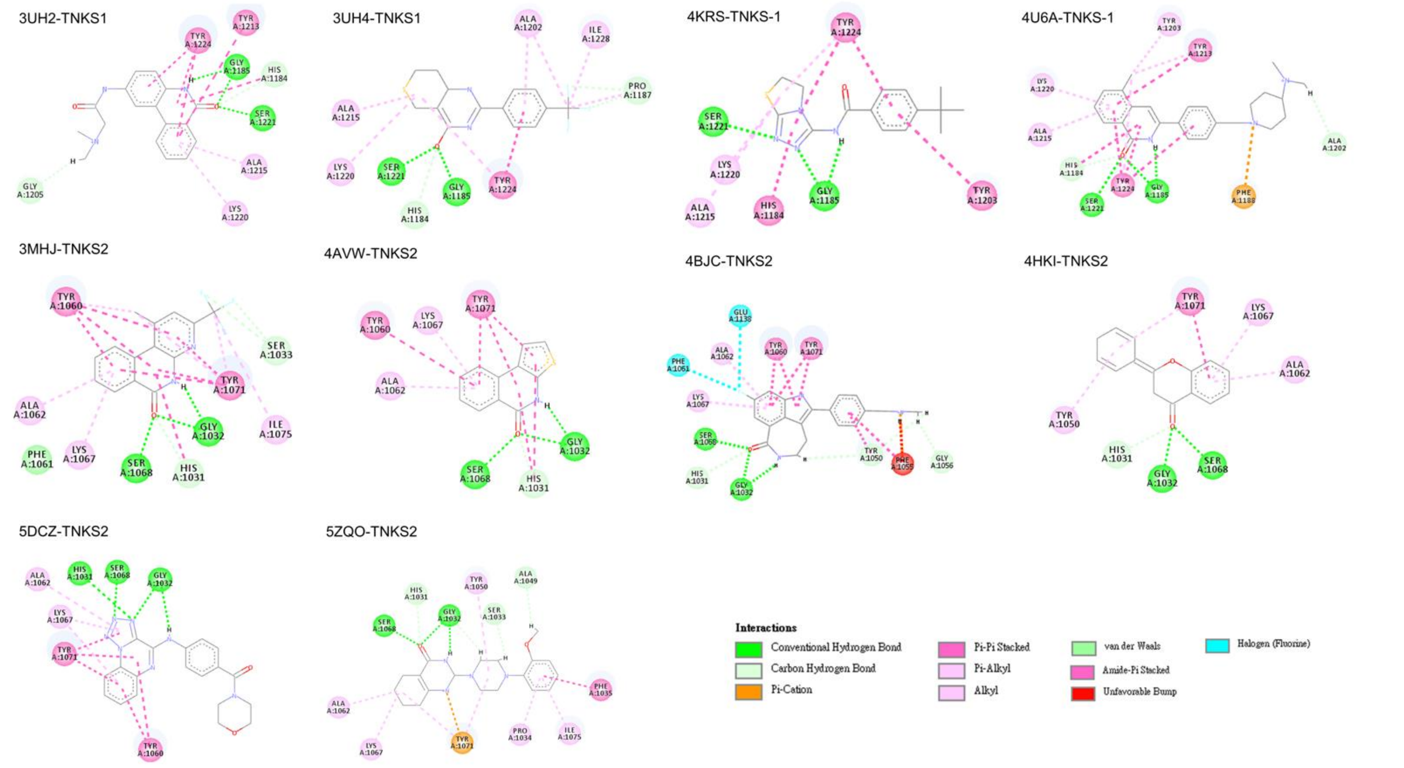

Figure S1. Detailed interaction maps of 10 co-crystal ligands with the surrounding residues of their crystal structures.

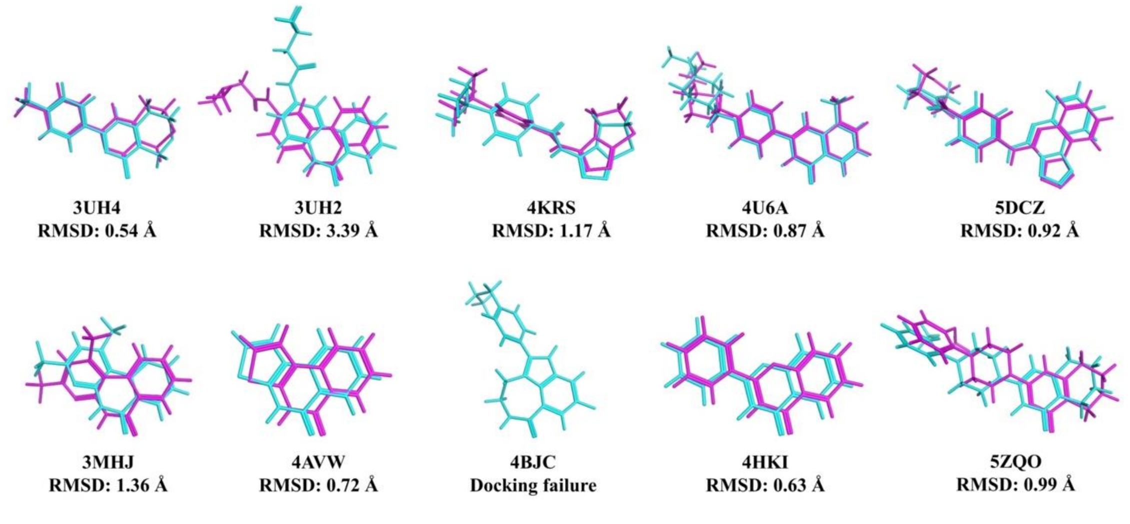

Figure S2. Redocking co-crystal ligand by pharmacophore docking. The cyan is the crystal structure, and purple is the docking pose.

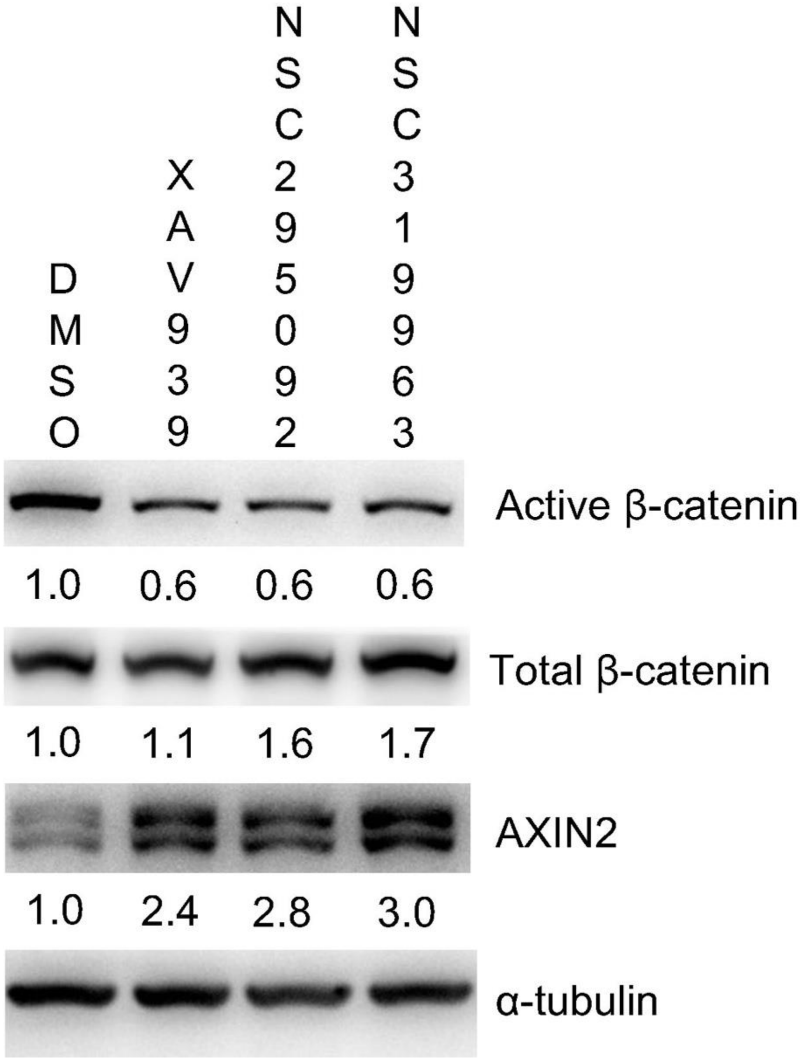

Figure S3. Cell lysates were collected at 48 hr and subjected to western blot for detection of active β-catenin, total β-catenin and AXIN2. α-tubulin was used as a loading control. The density of each band was normalized with α-tubulin by Image J software and presented as fold number between band space.

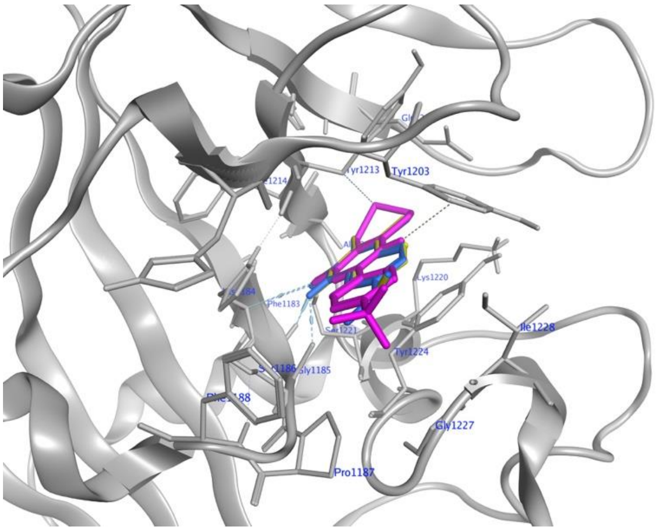

Figure S4. The superposition of compound XAV939 with screened compounds NSC319963 and NSC295092 in the binding pocket of TNKS-1. The binding poses are quite similar. Gray color is TNKS-1; purple color is compound XAV939; yellow color is compound NSC295092; blue color is compound NSC319963.

Here are the loading sequences of the original western blot images.

α-tublin

Last four bands are non-related experiments.

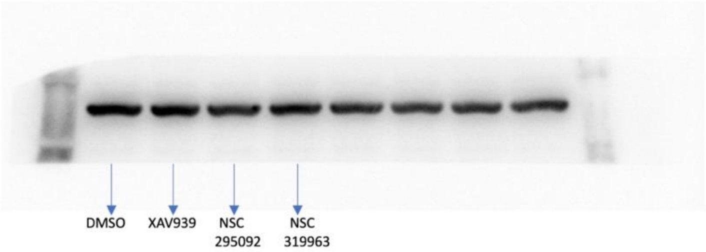

AXIN2

Last four bands are non-related experiments.

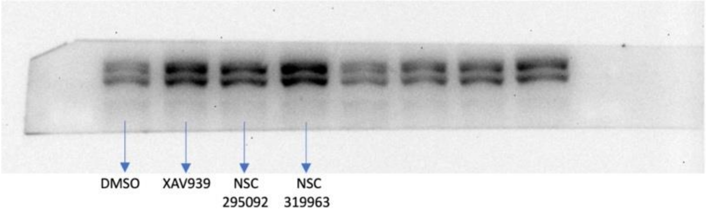

Total β-catenin

First four bands are non-related experiments.

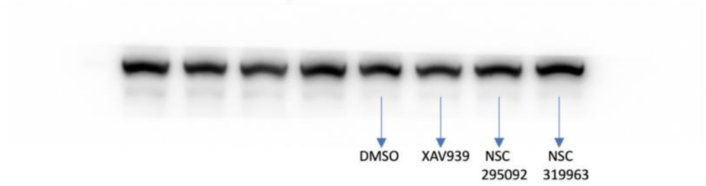

Active β-catenin

Last four bands are non-related experiments.

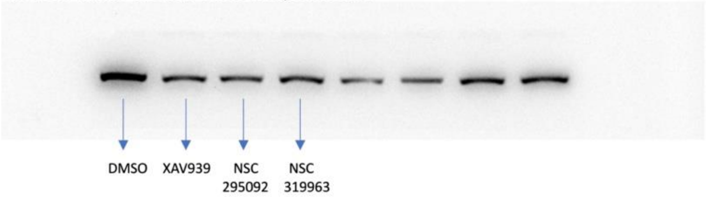

Supplement: Supplementary file 1 [file biomedicines-10-00143-s001.zip › biomedicines-1512958-supplementary.pdf]
